# Math on cortex—enhanced delta phase synchrony in math experts during long and complex math demonstrations

**DOI:** 10.1093/cercor/bhae025

**Published:** 2024-02-13

**Authors:** Hanna Poikonen, Samuel Tobler, Dragan Trninić, Cléa Formaz, Venera Gashaj, Manu Kapur

**Affiliations:** Professorship for Learning Sciences and Higher Education, Department of Humanities, Social and Political Sciences, Swiss Federal Institute of Technology (ETH) Zurich, Zurich 8092, Switzerland; Centre of Excellence in Music, Mind, Body and Brain, Faculty of Educational Sciences, University of Helsinki, Helsinki 00014, Finland; Professorship for Learning Sciences and Higher Education, Department of Humanities, Social and Political Sciences, Swiss Federal Institute of Technology (ETH) Zurich, Zurich 8092, Switzerland; Professorship for Learning Sciences and Higher Education, Department of Humanities, Social and Political Sciences, Swiss Federal Institute of Technology (ETH) Zurich, Zurich 8092, Switzerland; Professorship for Learning Sciences and Higher Education, Department of Humanities, Social and Political Sciences, Swiss Federal Institute of Technology (ETH) Zurich, Zurich 8092, Switzerland; Professorship for Learning Sciences and Higher Education, Department of Humanities, Social and Political Sciences, Swiss Federal Institute of Technology (ETH) Zurich, Zurich 8092, Switzerland; Department of Psychology, University of Tuebingen, Tuebingen 72076, Germany; Professorship for Learning Sciences and Higher Education, Department of Humanities, Social and Political Sciences, Swiss Federal Institute of Technology (ETH) Zurich, Zurich 8092, Switzerland

**Keywords:** EEG, Expertise, Mathematics, Complex cognition, Body posture

## Abstract

Neural oscillations are important for working memory and reasoning and they are modulated during cognitively challenging tasks, like mathematics. Previous work has examined local cortical synchrony on theta (4–8 Hz) and alpha (8–13 Hz) bands over frontal and parietal electrodes during short mathematical tasks when sitting. However, it is unknown whether processing of long and complex math stimuli evokes inter-regional functional connectivity. We recorded cortical activity with EEG while math experts and novices watched long (13–68 seconds) and complex (bachelor-level) math demonstrations when sitting and standing. Fronto-parietal connectivity over the left hemisphere was stronger in math experts than novices reflected by enhanced delta (0.5–4 Hz) phase synchrony in experts. Processing of complex math tasks when standing extended the difference to right hemisphere, suggesting that other cognitive processes, such as maintenance of body balance when standing, may interfere with novice’s internal concentration required during complex math tasks more than in experts. There were no groups differences in phase synchrony over theta or alpha frequencies. These results suggest that low-frequency oscillations modulate inter-regional connectivity during long and complex mathematical cognition and demonstrate one way in which the brain functions of math experts differ from those of novices: through enhanced fronto-parietal functional connectivity.

## Introduction

Rhythmic fluctuations in neural activity distribute throughout the brain over a wide spectrum of frequencies (Buzsáki 2006). Such oscillatory activity plays a critical role in neural processing and information transfer between near and far brain regions (Buzsáki and Draguhn 2004). Brain oscillations seem to be an underlying mechanism in perceptual and cognitive processes (Başar et al. 2001), and further, these processes can be modified by expertise (Grabner et al. 2006; Puschmann et al. 2021).

Since neural oscillations on several frequencies underlie information transfer in the brain locally and globally, their function is in specific interest during challenging cognitive tasks, like mathematical processing. Many mathematicians argue that elementary arithmetic presents only a tiny subset of the variety of domains that mathematics encompasses (Amalric and Dehaene 2017). Recently, advanced mathematics and math expertise has been studied through hemodynamic changes in the brain via functional magnetic resonance imaging (fMRI; Amalric and Dehaene 2017 for a review; Wang et al. 2022). These studies agree with the activation of the visuospatial network when processing math tasks supporting visuospatial representation and mental manipulation of abstract symbols and relations. Intraparietal and inferior temporal regions seem to form the core of this network activating even when judging simple and overlearned mathematical facts, whereas frontal regions are primarily called upon during intense and prolonged math reflections (Amalric and Dehaene 2016).

Another network likely involved in advanced mathematical processing is the multiple-demand system which is active during various difficult cognitive tasks involving executive control (Duncan 2010). The core of such network includes posterior-medial frontal cortex, bilateral anterior insula, intraparietal sulcus and posterior inferior frontal sulcus (Duncan 2010; Müller et al. 2015). A recent study mapped and characterized an extended multiple-demand system including several regions which are in close interaction with the core network (Camilleri et al. 2018). These results highlight the relevance of several sub-networks each of which subserves specific roles within the multiple-demand system (Camilleri et al. 2018). Taken together, interaction of the multiple-demand and visuospatial networks over the fronto-parietal regions may enable processing of advanced mathematics.

Electroencephalography (EEG) studies have shown that engaging with short artihmetic tasks modifies brain oscillations (De Smedt et al. 2009; Grabner and De Smedt 2012; Mosbacher et al. 2020). More specifically, training in arithmetic strategies modified theta (3–6 Hz) and lower alpha (8–10 Hz) amplitude synchronies over parieto-occipital electrodes (Grabner and De Smedt 2012). Arithmetic fact retrieval is associated with theta amplitude synchrony over left hemisphere, and procedural strategies with parieto-occipital alpha amplitude desynchrony (De Smedt et al. 2009). Further, frontal and parietal alpha and theta amplitude synchrony during short arithmetic tasks can be modified by pre-stimulus transcranial direct current stimulation influencing training gains (Mosbacher et al. 2020).

Some studies have sought to identify lower oscillations involved in mathematical processing by examining rhythmic fluctuations in the delta band (0.5–4 Hz) over the frontal and parietal electrodes (Harmony et al. 1996, 1999; Dimitriadis et al. 2010). These studies used a similar amplitude power method as the abovementioned studies over theta and alpha frequencies. However, these amplitude power methods measured over single electrode cannot provide a link between the large-scale inter-regional neural oscillations potentially underlying mathematical cognition, as suggested by the recent fMRI studies (Amalric and Dehaene 2016, 2019; Wang et al. 2022). However, the results by Harmony and colleagues, (1996, 1999) and Dimitriadis et al. (2010) indicate that rhythmic processes over delta frequencies are involved during mathematical processing. These lower delta frequencies are suggested to reflect internal concentration, which is required during cognitively demanding tasks such as mental calculation (Harmony 2013). It remains unknown whether these delta synchronies during mathematical cognition extend to connect the distant fronto-parietal regions (Amalric and Dehaene 2016; Sokolowski et al. 2017 for a meta-analysis).

Recent studies aiming to understand brain processes underlying expertise and advanced mathematics have used relatively short stimuli with durations up to a few seconds (Amalric and Dehaene 2016, 2019; Wang et al. 2022). These studies can be associated with the tradition of reductionist laboratory studies which are highly controlled in terms of body position of the participants and brain processes related to short stimuli. Another emerging branch of brain research utilizes real-world like, so called naturalistic, paradigms to test the validity of the models, which are derived from highly controlled experiments, in real-world contexts. The results of the reductionist studies seldomly extend to the real-world settings whereas naturalistic studies evoke several simultaneous sensory, cognitive, emotional and embodied responses leaving the results open to multiple interpretations (Sonkusare et al. 2019; Cantlon 2020; Nastase et al. 2020; Zhang et al. 2021). For example, analyses of EEG data show that regions in frontal cortex activate selectively for stimuli that are coherent over longer time periods requiring slow accumulation of information (Honey et al. 2012). Regions within frontal and temporal regions show inter-subject correlation at low frequency bands whereas visual cortex shows such correlation at higher frequencies. These results suggest that short time scales are encoded in high frequencies and long time scales in lower carrier frequencies (Kauppi et al. 2010). As described above, demanding cognitive tasks, which require deeper internal concentration, enhance low-frequency synchrony on the delta band (Harmony 2013). Therefore, when investigating the brain functions during advanced mathematics which require processing of contextual information over long time scales, it is plausible to expand the frequency bands of interest to inter-regional lower delta frequencies in addition to the conventionally studied intra-regional alpha and theta synchronies.

All brain imaging studies discussed above are conducted when sitting (e.g. Grabner et al. 2006; Artemenko et al. 2019; Molina del Río et al. 2019) or lying on back (Amalric and Dehaene 2016, 2019; Wang et al. 2022). However, a recent study suggested that body posture may play a role in cognitive processing (Dodwell et al. 2019). These results showed that the visuospatial working memory functions faster and more accurately when standing than sitting. Standing up is a simple way to increase the cognitive arousal state in comparison to sitting, one of the simple measures being faster heartbeat when standing in comparison to sitting. Young et al. (2017) studied the activation and interaction of three large neural networks in the brain, default mode, executive control and salience network, in correlation with the arousal state measured with heartrate (faster the heartrate, higher the arousal state). The default mode network, the network of introspection and dreamy mind wandering, was shown to activate with slow heartrate. In contrast, the interaction of the executive control network, the network recruited during cognitively demanding tasks, and salience network, the network which helps to guide the attention towards that what is significant—both being included in the extended multiple-demand system—was shown to follow an inverted U shape and was the strongest during a moderately aroused state (Young et al. 2017; Camilleri et al. 2018).

Sitting or lying in a dark isolated laboratory, as in the conventional studies on mathematical cognition, may make participants tired enhancing the activation of the default mode network (Young et al. 2017). Thus, increased arousal state when standing, in comparison to sitting or lying, may dampen down the activity of the default mode network and enhance the function of the extended multiple-demand network, the recruitment of which is likely needed during advanced mathematics (Young et al. 2017; Schmitt et al. 2019).

To complement the hemodynamic studies on expertise and advanced mathematics (Amalric and Dehaene 2017 for a review; Wang et al. 2022), we designed an EEG study to investigate the inter-regional cortical synchronies in math experts and novices when they watched advanced (bachelor-level) math demonstrations over a long time span (up to 1 minute), and the influence of body posture in such processing. We were interested in relative expertise by taking two snapshots of expertise from the novice-expert continuum: University students in math and math-related fields, like physics or engineering (math experts), and participants without university-level math studies (novices).

We hypothesized that the fronto-parietal connections, which are previously associated with mathematical processing (Amalric and Dehaene 2016; Sokolowski et al. 2017 for a meta-analysis), activate more strongly in experts than novices during long advanced mathematics (Hypothesis 1). Therefore, we expected to observe an enhanced low-frequency functional connectivity between frontal and parietal electrodes in experts in comparison to novices. These results would suggest enhanced simultaneous and interactive domain-general (multiple demand system) and domain-specific (visuospatial network) processing in experts, which emerge in the frontal and parieto-temporal cortical regions (Camilleri et al. 2018; Pinheiro-Chagas et al. 2018; Amalric and Dehaene 2019; Wang et al. 2020). Alternatively, if experts relied solely on domain-specific mathematical processes, we might observe only stronger parietal phase synchrony in experts in comparison to novices (Amalric and Dehaene 2019). However, we estimated the difficulty level of the math tasks to be so high, that the experts would recruit both frontal and parietal processing (Amalric and Dehaene 2016). To our knowledge, the influence of sitting and standing body posture on the processing of long and complex cognitive tasks has not been investigated yet. Therefore, we aimed to explore the influence of standing body posture in advanced mathematical processing. Our explorative hypothesis (Hypothesis 2) was that a standing posture increases the state of arousal, and therefore, enhances differently in experts and novices the functions of the extended multiple-demand system, which is required during demanding cognitive tasks and functions optimally in moderately aroused states (Young et al. 2017; Camilleri et al. 2018). Our study is pioneering work in investigating the inter-regional cortical connectivity of math experts and novices during long advanced mathematics and the influence of body posture has on the brain processes during such complex cognition.

## Materials and methods

### Participants

Thirty-four math experts (bachelor and master students in math or math-related disciplines, like physics or engineering) and thirty-five math novices (completed high-school but no university-level math studies) participated in the experiment. However, eleven participants from the group of math experts and twelve participants from the novice group were discarded from the data analysis because either their EEG data was noisy due to heavy contamination by the 50 Hz line noise, or some of the relevant data was missing due to malfunctioning EEG amplifier. Thus, in the group of math experts, there were 22 participants (5 female and 17 male), and 22 participants in the novice group (7 female and 15 male). The background of the participants was screened by a math questionnaire. We were not able to calculate the sample size needed as there were no prior EEG studies on change in phase synchrony during long math demonstrations, and therefore no information on expected variability. We relied on the previous neuroscientific literature related to expertise of mathematical processing and we collected data from a similar number of participants (Grabner and De Smedt 2012; Amalric and Dehaene 2016; Jeon et al. 2019).

The age of the participants ranged from 19 to 24 years (mean 21.0 years) among math experts and from 19 to 35 years (mean 23.8 years) among novices. We aimed for age-matched groups. However, due to rejection of several participants, the age of the expert and novice groups differed statistically according to 1-way ANOVA: F(1,42) = 8.76, *P* = 0.005. All participants in both groups were right-handed. No participants reported hearing loss or history of neurological illnesses. The experiment protocol was conducted in accordance with the Declaration of Helsinki and approved by the Executive Board of ETH Zurich after a review by the ETH Zurich Ethics Commission. All participants provided written informed consent.

### Stimuli and procedure

**Fig. 1:**
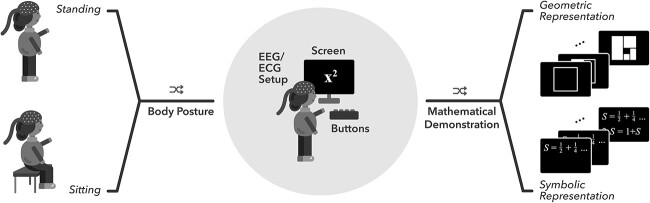
Study setup for participants watching symbolic and geometric math demonstrations in an EEG laboratory when sitting and standing.

**Fig. 2:**
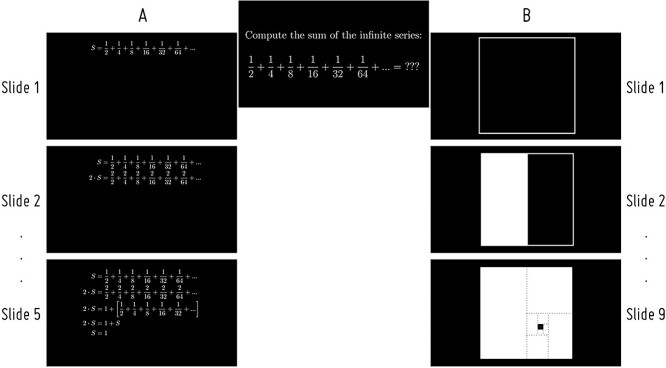
One out of 8 math demonstrations shown in symbolic (A) and geometric (B) form. The symbolic presentation of this specific math demonstration had in total 5 slides and the geometric presentation 9 slides. The slides 4 and 5 for the symbolic presentation are not shown in this image, nor the slides 3–8 for the geometric presentation. The first slide, the introduction of the math demonstration (image in the middle) was the same in both symbolic and geometric presentation and was not included in the analysis.

Participants watched in total 16 math demonstrations (8 demonstrations presented in symbolic and geometric manner), and after each demonstration they were asked four self-evaluation reflections to which they answered by pressing a button in a 4-button response box. Each set of trials contained 4 excerpts of the same presentation style (symbolic or geometric) in two conditions (sitting or standing), and these sets were presented in a pseudo random order via a monitor (Fig. 1). The pseudo randomization defined the presentation order (symbolic first or geometric first) and the order of body posture (sitting first or standing first). However, each participant saw the same four math demonstrations presented in both symbolic and geometric form before changing the body posture for the remaining set of four demonstrations presented in symbolic and geometric form. One out of 8 math demonstrations is shown in Fig. 2, both in a symbolic (Fig. 2A) and in a geometric form (Fig. 2B). The self-evaluation reflections (SER), which were presented after each demonstration, were the following: *I had enough of time to follow the math demonstration* (SER1), *I was familiar with the math demonstration* (SER2), *I understood the math demonstration* (SER3) and *I found this math demonstration engaging* (SER4). The options for the answers given via the response box were the following: 1 = *Completely disagree*, 2 = *Somewhat disagree*, 3 = *Somewhat agree*, and 4 = *Completely agree*. Self-evaluation reflections are an important indicator how well the participants understood the math demonstrations (SER3) and how deeply they felt engaged with each demonstration (SER4). In addition, we screened the familiarity (SER2), and whether the participants had sufficiently time to follow the steps of the demonstrations (SER1).

Each math demonstration consisted of several slides, varying from 4 to 12 slides (6.9 slides on average) depending on the complexity of the demonstration. The total duration of math demonstrations varied from 13 seconds to 68 seconds (33.1 seconds on average). The timing of each slide was fixed and the same for all the participants. The duration of each slide was defined according to a screening made online in which 25 math experts and 25 math novices watched the slides and went forward to a following slide with a button press. The participants who attended the online screening did not attend the actual experiment. The duration of each slide was the average time the participants spent on each slide. In the online screening, there was no statistically significant difference in the duration of time spent on each slide between experts and novices. If we had tried to match the duration of the stimuli, we might have presented the math demonstrations either to quickly or too slowly which would have contradicted with our aim to study the cortical processing underlying mathematical processing. Instead, we might have collected EEG data under confusion or boredom. Also, it would have been a somewhat impossible task to find a set of math demonstrations for which the time to solve them would be the same.

In addition, participants filled the Abbreviated Math Anxiety Scale (AMAS) and made a shorter version of the Berlin Intelligence Structure Test (BIS-T; Jäger et al. 1997) with a duration of 60 minutes. BIS-T is shown to measure well fluid intelligence (Beauducel and Kerstin 2002) which may influence in the function of the multiple-domain system in the brain (Duncan 2010). BIS-T was translated from German to English by our research group. In addition to general intelligence, it tests three components: Verbal, numeric and figural intelligence. It also tests four abilities influencing intelligence: Processing capacity, processing speed, memory retention and richness of ideas. AMAS was filled before watching the math demonstrations, and BIST-T as the last task of the experiment after the math demonstrations. Before the presentation of the math demonstrations, the baseline EEG data was collected from the participants in which they spent 120 seconds in each of the following states: sitting eyes closed, sitting eyes open, standing eyes closed and standing eyes closed.

The stimuli were presented to the participants with the MATLAB via PsychToolbox. The playback of the presentation program was launched by the researcher after which participant could navigate to the math demonstrations by a button press once they had read the instruction slides on the screen. The total length of the experiment material was approximately 15 minutes.

The data were recorded using Ant Neuro eego mylab (https://www.ant-neuro.com/products/eego_mylab) electrode caps with active 128 EEG channels and four external electrodes placed below, above and on the left side of the left eye and on the right side of the right eye. The offsets of the active electrodes were kept below 30 mv at the beginning of the measurement, and the data were collected with a sampling rate of 2048 Hz. The beginning of each slide of the math presentations were marked with a trigger into the EEG data, as well as the button presses related to the instructions and the self-evaluation questions. The triggers were sent wirelessly via Lab Streaming Layer (LSL; RRID:SCR 017631, https://github.com/sccn/labstreaminglayer). In addition to the EEG data, also bipolar electrocardiography (ECG) was collected by placing one sensor below the right collar bone and another sensor under the lowest left rib. The EEG and ECG data were synchronized.

### Data analysis

#### Preprocessing

The EEG data of all the participants were first preprocessed with EEGLAB (version 2019.1; Delorme and Makeig 2004). The average of all the EEG electrodes was set as a reference. The data were high-pass filtered at 0.5 Hz and low-pass filtered at 40 Hz. Finite impulse response (FIR) filtering, based on the firls (least square fitting of FIR coefficients) MATLAB function, was used as a filter for all the data. The data were then treated with independent component analysis (ICA) decomposition with the runica algorithm of EEGLAB (Delorme and Makeig 2004) to detect and remove artifacts related to eye movements and blinks. ICA decomposition gives as many spatial signal source components as there are channels in the EEG data. Typically, one to four ICA components related to the eye artifacts were removed. Noisy EEG data channels for some participants were interpolated. After the interpolation, the data were split into the frequency bands of 0.5–4 Hz (delta), 4–8 Hz (theta) and 8–13 Hz (alpha) with high-pass and low-pass filtering. In addition, these frequency bands were extracted for 90 seconds of rest EEG (sitting eyes closed, sitting eyes open, standing eyes closed, standing eyes open) starting from 15 seconds after the participant had started each resting state task.

### Procedure of the synchrony analyses

We calculated the phase synchrony values (PSVs) of the EEG data to the math demonstrations. More in detail, the PSV value was calculated for 5-second segments with 50% overlapping for each math demonstration excluding the first two seconds and last one second of the presentation (referred as PSV_WholeStimuli). For example, if the duration of the math demonstration was 54 seconds, the PSV value was calculated for the period of 2–53 seconds divided to 5-second segments with an overlapping of 2,5 seconds in between the sequential segments. Since the first 2 seconds were not included to the abovementioned analysis, and the brain tends to habituate to ongoing stimuli, we also analyzed separately first 250–2000 ms with 500 ms segments with 50% overlapping (referred as PSV_FirstTwoSeconds). Also, previous EEG studies in math generally use shorter stimuli than the ones we used, with a duration of a couple of seconds. In addition, during the first 2 seconds, in all the demonstrations only the first slide of the math demonstrations was presented. Thus, we were interested in studying separately 1) the beginning of stimuli to avoid habituation and study a similar duration as in previous EEG studies, and 2) the whole stimuli to study the brain processes during complex math with long duration.

The PSV was calculated separately for both PSV_WholeStimuli and PSV_FirstTwoSeconds based on the Hilbert transformation of the phases of the data stream by an electrode pair under comparison. The Hilbert-based method, introduced by Tass et al. (1998), is widely used in phase synchrony analysis (e.g. Hong et al. 2006; Wang et al. 2006). A similar method has also been used in EEG data analysis with continuous music stimuli (Bhattacharya and Petsche 2000; Poikonen et al. 2018a) and dance stimuli (Poikonen et al. 2018b).

For PSV_WholeStimuli and PSV_FirstTwoSeconds, we conducted the synchrony analysis over the 12 electrodes of F3 (5), Fz (6), F4 (7), FCz (42), Cz (16), CP3 (48), CP4 (49), P1 (51), Pz (26), P2 (52), PPO1 (92) and PPO2 (93; the 128-channel Ant Neuro EEG gap; Fig. 1) so that each electrode was compared pairwise to all the other ones resulting in 66 electrode pairs of comparison. These electrodes were chosen on both hemispheres and the central line along the fronto-parietal pathway based on the previous literature on math expertise (Grabner et al. 2007; Amalric and Dehaene 2016; Sokolowski et al. 2017; Camilleri et al. 2018) and multiple-demand system (Duncan 2010; Camilleri et al. 2018). We were also interested in the influence of early visual regions in mathematical processing (DeWind et al. 2019). Oftentimes, the occipital electrodes are noisy due to thick layers of hair or neck muscle activity. Because of this practical reason, we chose to include two parieto-occipital electrodes into our analysis instead of the occipital electrodes. Channel CPz was the reference electrode in our cap configuration and could not be used in the data analysis. Some of the participants had individual channels with bad signal, and thus, were interpolated based on the surrounding channels. Such procedure was made to maximum one participant in each group. Bad channels were interpolated for channel Fz (Expert22 and Expert 73) and F4 (Expert20). The analysis was made separately for each frequency band of delta, theta and alpha. All the PSVs of the 5-s segments were first averaged for each math demonstration of each participant, and then, over each stimulus condition (four math demonstrations in each: Symbolic-Sitting, Geometric-Sitting, Symbolic-Standing, and Geometric-Standing) of each participant. Thus, for each condition, each participant got a unitary PSV for each electrode pair. In addition, PSV was calculated over the 75-second rest EEG data, during which the participant first sat quietly eyes open, and then stood still eyes open, in a dark and silent EEG laboratory. Also, PSV was calculated over the 75-second rest EEG data with eyes closed when sitting and standing. Similarly to the stimulus data, the data of the rest EEG were segmented with 5 seconds and 50% overlapping of the two consecutive segments separately for the eyes closed sitting, eyes open sitting, eyes closed standing and eyes open standing conditions. For each participant, the rest PSVs calculated for the 5-s segments were averaged as a unitary PSV value for each electrode pair individually for each these four conditions.

### Statistical analyses

The statistical analyses were conducted with MATLAB version R2019b. The repeated-measures ANOVA with between-subject factor Group (Math Experts and Novices) and within-subject factors Posture (Sitting Eyes Open and Standing Eyes Open for the resting state and Sitting and Standing for math demonstrations, and Stimulus: Symbolic and geometric math demonstration), was conducted separately for each electrode pair (66 electrode pairs) of PSV_FirstTwoSeconds and PSV_WholeStimulus, each frequency band (delta, theta and alpha) and both during math demonstrations and rest. The main effects for the factor Group, Posture and Stimulus, and the Group*Posture, Group*Stimulus interactions were calculated with the Greenhouse–Geisser (GG) adjustment. In Results, these results are referred to with pGG indicating the Greenhouse–Geisser adjusted p-values of the repeated-measures ANOVA.

The multiple comparisons of Group, Posture and Stimulus were calculated with the critical value of Bonferroni. In Results, p indicates the p values of these multiple comparisons of Group, Posture and Stimulus. For PSVs, the comparison of 66 electrode pairs increased the Type 1 error. Thus, false discovery rate (FDR) was calculated for each set of 66 electrode pairs of PSV_FirstTwoSeconds and PSV_WholeStimulus from their pGG values of the results of the repeated-measures ANOVA to control the expected proportion of false positives.

## Results

In this paper, we focus on reporting and discussing the influence of expertise in naturalistic mathematical processing.

### Behavioral results

**Fig. 3:**
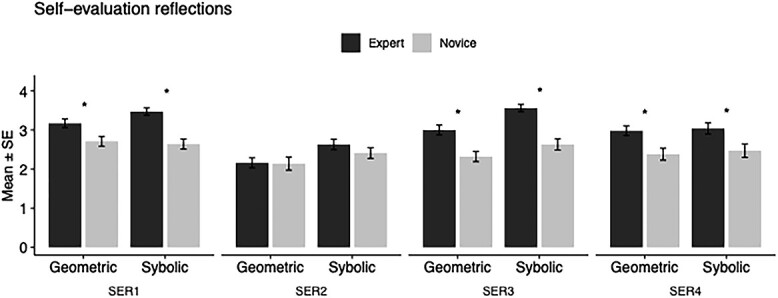
Self-evaluation reflections 1–4 of math experts (black bars) and novices (light gray bars) with error bars of standard error. Statistically significant group differences are marked with an asterisk *. The self-evaluation reflections (SER), which were presented after each demonstration, were the following: *I had enough of time to follow the math demonstration* (SER1), *I was familiar with the math demonstration* (SER2), *I understood the math demonstration* (SER3) and *I found this math demonstration engaging* (SER4).

Self-evaluation reflection. Repeated measures ANOVA was calculated individually for each SER with between factor Group (Experts, Novices) and within-factors Stimulus (Symbolic, Geometric) and Posture (Sitting, Standing). The SERs were the following: *I had enough of time to follow the math demonstration* (SER1), *I was familiar with the math demonstration* (SER2), *I understood the math demonstration* (SER3) and *I found this math demonstration engaging* (SER4). SER1, SER3 and SER4 results differed significantly between experts and novices, and they are presented in Table 1 and Fig. 3.

**Table 1:** Repeated measures ANOVA was calculated individually for each self-evaluation reflection with between factor group (experts, novices) and within-factors stimulus (symbolic, geometric) and posture (sitting, standing). There were no statistically significant interactions for group*stimulus nor group*posture.

Self-evaluation reflection (SER)	F(1,42)	pGG	Multiple comparison (Bonferroni)	Mean value Experts (standard deviation)	Mean value Novice (standard deviation)
SER1: *I had enough of time to follow the math demonstration*	Group: 25.19 Stimulus: 0.36 Posture: 0.095	**Group: 1.00e-05** Stimulus: 0.55 Posture: 0.76	Experts > Novices, *P* = 1.00e-05	3.35 (0.56)	2.75 (0.71)
SER2: *I was familiar with the math demonstration*	Group: 0.012 Stimulus: 19.17 Posture: 0.060	Group: 0.91 **Stimulus: 7.78e-05** Posture: 0.8075	Symbolic > Geometric, *P* = 7.78e-05	2.37 (0.82)	2.35 (0.81)
SER3: *I understood the math demonstration*	Group: 24.24 Stimulus: 35.74 Posture: 1.72	**Group: 1.36e-05** **Stimulus: 4.27e-07** Posture: 0.20	Experts > Novices, *P* = 1.36e-05 Symbolic > Geometric, *P* = 4.27e-07	3.33 (0.62)	2.56 (0.71)
SER4: *I found this math demonstration engaging*	Group: 7.46 Stimulus: 0.12 Posture: 3.38	**Group: 0.0092** Stimulus: 0.73 Posture: 0.073	Experts > Novices, *P* = 0.0092 Sitting > Standing, *P* = 0.073 (marginally significant)	3.03 (0.67)	2.53 (0.77)

Math Anxiety. The one-way ANOVA analysis of the Abbreviated Math Anxiety Scale (AMAS; Hopko et al. 2003) revealed no statistically significant differences between math experts (mean = 18.48; SD = 4.20) and math novices (mean = 19.86; SD = 6.70) regarding math anxiety (F(1,41) = 0.65, *P* = 0.42).

Intelligence. Using a repeated-measures ANOVA approach with the *between-factor* Group (math experts or math novices) and *within-factors* Intelligence Components (verbal IQ, numerical IQ, figural IQ, total IQ, and cognitive tilt between verbal and numerical IQ), we found no statistically significant differences for the Group comparison (F(1,41) = 0.71, *P* = 0.40), nor for the Group*Intelligence Component interaction (F(4,164) = 0.50, *P* = 0.74). A further repeated-measures ANOVA was conducted to investigate differences *between* the Groups (math experts and math novices) and *within* the Intelligence Ability (processing capacity, processing speed, memory retention, richness of ideas). There were no statistically significant differences for Group (F(1,41) = 0.80, *P* = 0.38) and for the Group*Intelligence Ability interaction (F(3,123) = 1.75, *P* = 0.16).

### E‌EG results

**Fig. 4:**
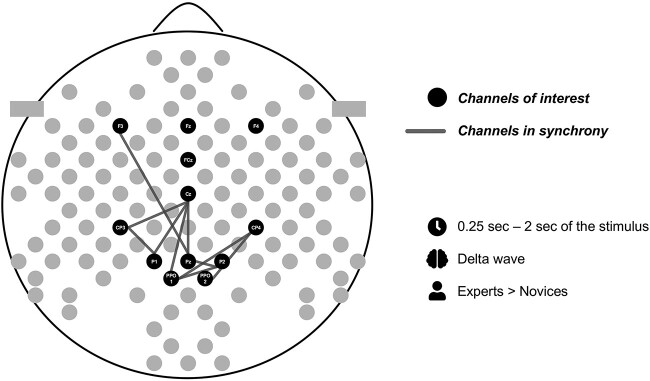
Electrode pairs with significant group differences for 0.25–2 seconds from the stimulus onset.

**Fig. 5:**
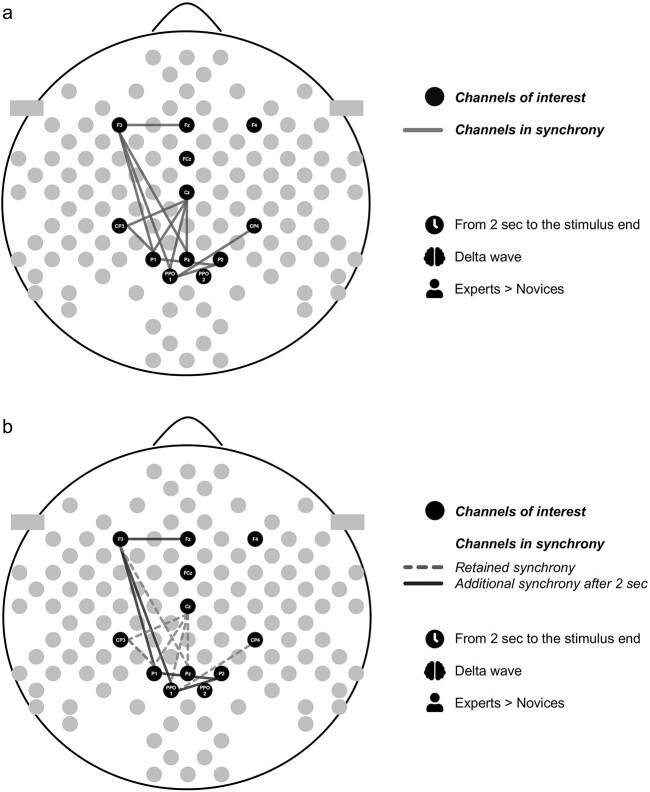
a). Electrode pairs with significant group differences from 2 seconds of the stimulus onset to the end of the stimulus excluding the last second. b). Comparison of the statistically significant group differences in delta phase synchrony over the electrode pairs during the first two seconds from the stimulus onset and from two seconds of the stimulus onset to the end of the stimulus.

**Fig. 6:**
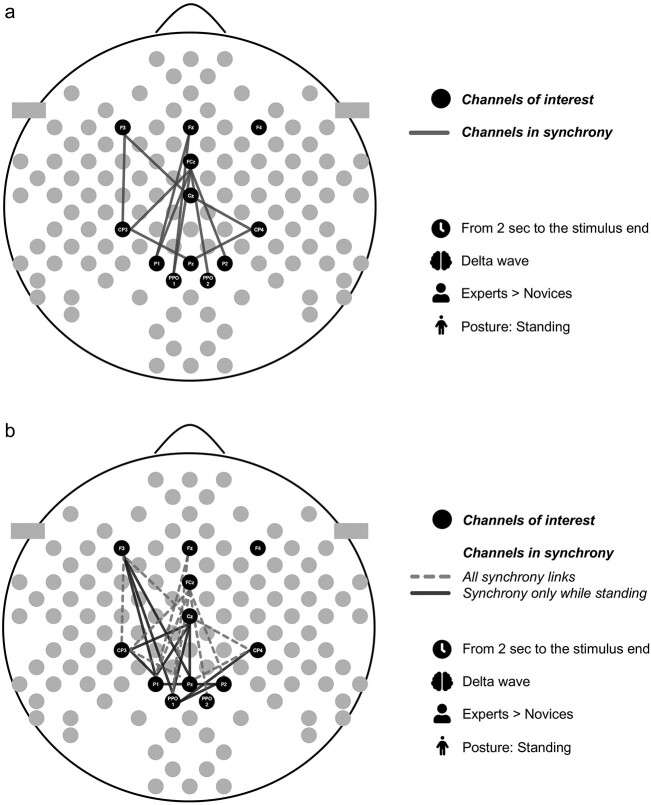
a). Electrode pairs with significant group*posture interactions for standing condition from 2 seconds of the stimulus onset to the end of the stimulus excluding the last second. b). Comparison of the statistically significant group differences in delta phase synchrony over the electrode pairs during both body postures, sitting and standing, and during standing only.

The only statistically significant Group differences and their interactions occurred over the delta band, and they are reported in Tables 2 and 3. The content of Table 2 is illustrated in Fig. 4 and Table 3 in Figs. 5 and 6. The electrode pairs reported did not have statistically significant Group differences during the baseline data (sitting and standing eyes open). According to the repeated-measures ANOVA, there were some more significant Group*Condition interactions. However, after applying the multiple comparison with the critical value of Bonferroni, these results did not remain significant, and thus, are not reported. On higher frequency bands—theta and alpha—there were no statistically significant Group differences, and thus, results from those frequency bands are not reported.

**Table 2:** The significant results for the PSV_FirstTwoSeconds, calculated from 250 milliseconds to 2 seconds of the stimulus onset, are presented. On the delta band, there were several electrode pairs with significant group effect. The time window for the phase synchrony value is 0.5 seconds with 50% overlapping.

Delta: 0.5–4 Hz First 2 sec	Electrode pair	F(1,42)	pGG value	pFDR	Multiple comparison (Bonferroni)	Mean PSV Experts (standard deviation)	Mean PSV Novices (standard deviation)
Group main effect	F3—Pz	4.10	0.049	0.0017	Experts > Novices	0.71 (0.13)	0.67 (0.16)
	Cz – Pz	4.56	0.039	0.0022	Experts > Novices	0.76 (0.14)	0.66 (0.14)
	Cz – CP3	4.29	0.044	0.0019	Experts > Novices	0.75 (0.15)	0.66 (0.15)
	Cz – P1	4.73	0.035	0.0031	Experts > Novices	0.76 (0.14)	0.66 (0.16)
	Cz – PPO1	7.53	0.0089	0.0015	Experts > Novices	0.75 (0.13)	0.64 (0.13)
	Pz – P2	7.81	0.0078	0.0027	Experts > Novices	0.81 (0.14)	0.68 (0.17)
	CP3 – P1	4.96	0.031	0.0036	Experts > Novices	0.76 (0.15)	0.66 (0.15)
	CP4 – PPO1	4.16	0.048	0.0018	Experts > Novices	0.75 (0.15)	0.66 (0.14)
	P1 – P2	4.67	0.036	0.0025	Experts > Novices	0.78 (0.16)	0.68 (0.16)
	P2 – PPO1	4.41	0.042	0.0021	Experts > Novices	0.79 (0.15)	0.69 (1.18)

**Table 3:** The significant results for the PSV_WholeStimulus, calculated from 2 seconds of the stimulus onset to one second before the end of the stimulus, are presented. On the delta band, there were several electrode pairs with significant group effect and group*posture interactions. The time window for the phase synchrony value is 5 seconds with 50% overlapping.

Delta: 0.5–4 Hz From 2 sec onwards	Electrode pair	F(1,42)	pGG value	pFDR	Multiple comparison (Bonferroni)	Mean PSV Experts (standard deviation)	Mean PSV Novices (standard deviation)	pGG value of the same electrode pair in the baseline condition (sitting and standing eyes open)
Group main effect	F3 – Fz	5.88	0.020	0.0038	Experts > Novices	0.44 (0.13)	0.36 (0.14)	0.25
	F3 – Pz	5.54	0.023	0.0028	Experts > Novices	0.38 (0.11)	0.32 (0.11)	0.27
	F3 – P1	4.37	0.043	0.0028	Experts > Novices	0.38 (0.11)	0.33 (0.12)	0.27
	F3 – PPO1	4.72	0.036	0.0029	Experts > Novices	0.38 (0.11)	0.32 (0.11)	0.55
	Cz – Pz	6.02	0.018	0.045	Experts > Novices	0.42 (0.12)	0.35 (0.12)	0.75
	Cz – CP3	4.94	0.032	0.0028	Experts > Novices	0.41 (0.13)	0.35 (0.13)	0.98
	Cz – P1	5.78	0.021	0.0034	Experts > Novices	0.42 (0.13)	0.35 (0.13)	0.70
	Cz – P2	5.06	0.030	0.0029	Experts > Novices	0.41 (0.13)	0.34 (0.13)	0.93
	Cz – PPO1	8.78	0.0050	0.0049	Experts > Novices	0.42 (0.12)	0.34 (0.11)	0.35
	Pz – P2	8.61	0.0054	0.0026	Experts > Novices	0.45 (0.13)	0.36 (0.14)	0.48
	CP3 – P1	6.03	0.018	0.0059	Experts > Novices	0.42 (0.13)	0.35 (0.13)	0.96
	CP3 – PPO1	4.67	0.036	0.0027	Experts > Novices	0.42 (0.13)	0.35 (0.12)	0.52
	CP4 – PPO1	5.12	0.029	0.0031	Experts > Novices	0.41 (0.13)	0.35 (0.12)	0.93
	P1 – P2	4.52	0.039	0.0027	Experts > Novices	0.43 (0.14)	0.36 (0.13)	0.82
	P2 – PPO1	5.76	0.021	0.0029	Experts > Novices	0.44 (0.13)	0.36 (0.14)	0.85
**Delta:** **0.5–4 Hz** **From 2 sec onwards**	**Electrode pair**	**F(1,42)**	**pGG value**	**pFDR**	**Multiple comparison (Bonferroni)**	**Mean PSV Experts Standing (standard deviation)**	**Mean PSV Novices Standing (standard deviation)**	**pGG value of the same electrode pair in the baseline condition (standing eyes open)**
Group*Posture interaction	F3 – Cz	6.56	0.014	0.00060	Standing: Experts > Novices (*P* = 0.017)	0.40 (0.13)	0.33 (0.11)	0.15
	F3 – CP3	8.02	0.0071	0.0027	Standing: Experts > Novices (*P* = 0.039)	0.38 (0.13)	0.31 (0.11)	0.45
	Fz – P1	4.33	0.044	0.00040	Standing: Experts > Novices (*P* = 0.036)	0.38 (0.12)	0.31 (0.12)	0.34
	Fz – PPO1	5.23	0.027	0.00045	Standing: Experts > Novices (*P* = 0.028)	0.38 (0.13)	0.32 (0.10)	0.36
	Cz – CP4	4.82	0.034	0.00043	Standing: Experts > Novices (*P* = 0.023)	0.40 (0.13)	0.33 (0.11)	0.36
	Pz – CP3	4.12	0.049	0.00043	Standing: Experts > Novices (*P* = 0.020)	0.41 (0.14)	0.33 (0.11)	0.088
	Pz – CP4	4.34	0.043	0.00042	Standing: Experts > Novices (*P* = 0.024)	0.41 (0.15)	0.33 (0.11)	0.14
	FCz – CP3	6.37	0.016	0.00059	Standing: Experts > Novices (*P* = 0.027)	0.40 (0.13)	0.33 (0.11)	0.26
	FCz – P1	5.38	0.025	0.00050881	Standing: Experts > Novices (*P* = 0.049)	0.38 (0.13)	0.32 (0.13)	0.69
	FCz – P2	4.80	0.034	0.00039	Standing: Experts > Novices (*P* = 0.040)	0.38 (0.13)	0.31 (0.11)	0.83
	FCz – PPO1	4.80	0.034	0.00041	Standing: Experts > Novices (*P* = 0.020)	0.39 (0.13)	0.32 (0.10)	0.62
	FCz – PPO2	4.93	0.032	0.00043	Standing: Experts > Novices (*P* = 0.049)	0.38 (0.13)	0.32 (0.11)	0.62

## Discussion

We investigated whether math experts and novices show different inter-regional functional connectivity during long advanced mathematics, and whether differences exist in such connectivity when sitting or standing. We studied the functional connectivity with phase synchrony over the fronto-parietal electrodes and reasoned simultaneous and interactive fronto-parietal processes to be crucial during long advanced mathematical processing. We expected experts would show an enhanced fronto-parietal delta phase synchrony related to the coordination of the frontal processing, which is associated with intense and prolonged math reflection and activation of the multiple demand system, and parietal processing which is associated with the core knowledge of mathematics (Amalric and Deahene, 2016, 2019; Camilleri et al. 2018). Consistent with our Hypothesis 1, our results suggest that experts have a stronger fronto-parietal functional connectivity during long advanced mathematics in comparison to novices. These results were observed over the frontal and parietal electrodes in the left hemisphere which suggest the recruitment of the left-hemispheric language system included in the triple-code model of the number system (Dehaene 1992; Amalric and Dehaene 2017). Our explorative hypothesis (Hypothesis 2) was related to the expert-novice differences in the functional cortical connectivity and body posture. We found that experts had a stronger delta phase synchrony over bilateral fronto-parietal electrodes than novices when standing.

### Advanced mathematics and cortical connectivity

Our study aims to start bridging the gap in mathematical cognition which is often investigated through reductionist study designs but practiced in the real world. Reliance on traditionally used reductionist designs may lead to oversimplified and incomplete theories lacking real-world observations which are necessary to complete theories on mathematical cognition (Cantlon 2020). For example, simplified tasks, like number comparisons, seem to have a relatively limited causal role in math learning. Instead, versatile constellation of domain-general and domain-specific processes seem to play a role in such a learning process. In children, neural responses to real-world math are shown to predict better their math achievement than traditional reductionist number comparisons (Cantlon and Li 2013). In adults, increased complexity of mathematical tasks is shown to increase the activation of the frontal cortical regions (Amalric and Dehaene 2016), whereas the involvement of the semantic network is still under debate, and potentially related to the task complexity, duration and whether the brain imaging data is collected during the presentation of the math tasks or afterwards during the contemplation period (Amalric and Dehaene 2017; Wang et al. 2022). Thus, controlled short tasks on simple numerical representations may neutralize frontal processes, whereas longer and more advanced math tasks may enable dynamic interactions between multiple demand and domain-specific brain processes. Such dynamic interactions are impossible to capture with short and simple math tasks (Cantlon 2020).

Engagement with long and cognitively challenging stimuli, such as advanced mathematics, requires attention, active comprehension, and integration of information across multiple time scales (Sonkusare et al. 2019). Such long and complex stimuli are shown to activate the heteromodal association cortices (prefrontal, superior temporal and inferior parietal cortices) which refer to brain regions in charge of longer temporal dynamics of stimuli. Activity in these areas is not time-locked to low-level stimulus features but to the longer contextual narrative. Such temporal “receptive window” seems to differ from 12 to 36 seconds depending on the brain region (Hasson et al. 2008). Thus, in our study the difference in fronto-parietal functional connectivity between math experts and novices during long advanced mathematical processing may be driven by three interrelated aspects: experts’ deeper engagement with the demonstrations, their enhanced interaction between the multiple-demand and domain-specific cortical functions, and their modified heteromodal association brain processes related to contextual understanding of the demonstrations (Hasson et al. 2008; Harmony 2013; Amalric and Dehaene 2016; Camilleri et al. 2018).

### Delta synchrony during mathematical cognition

The enhanced delta synchrony that we observed in math experts is consistent with a previous result demonstrating wide-spread delta synchrony during math tasks (Dimitriadis et al. 2010). However, Dimitriadis et al. (2010) observed the result with amplitude synchrony. A disadvantage of this method is that amplitude is less robust for synchronies within a nonlinear system, like the brain (Breakspear 2002), and that it is limited to local intra-regional synchrony focusing on only one electrode at the time. The phase synchrony method, measured between two electrodes, is more robust to detect synchronies in a nonlinear system (Breakspear 2002). Two signals over distant electrodes may have different amplitudes and phases, whereas high coherence occurs when this phase difference remains. Therefore, we reasoned that the phase synchrony method is the most suitable for tracking cortical inter-regional functional connectivity, as aimed in our study.

To support the explanation that the enhancement in the fronto-parietal delta synchrony occurred because experts relied on simultaneous multiple-demand and domain-specific processes, it is important to rule out that the experts were more familiar with the math demonstrations (Grabner et al. 2009), they were more intelligent (Beauducel and Kerstin 2002; Grabner et al. 2006, 2007; Duncan 2010; Camilleri et al. 2018) or less anxious (Klados et al. 2017) than novices. These factors could influence in the low-frequency connectivity (Jaiswal et al. 2019). The self-evaluation reflections presented to the participants after each math demonstration revealed no difference in familiarity between experts and novices. Neither can we suggest the experts’ brains to function in a more efficient or connected manner due to higher intelligence since there were no differences in any IQ component (verbal, numeric, figural) or ability (processing capacity, processing speed, memory retention, richness of ideas). Neither there were group differences in math anxiety nor in resting state connectivity on the delta band. In contrast, we showed that, according to the self-evaluation reflections, experts were more engaged with the math demonstrations and understood them better than novices. Therefore, we reason that these differences on delta phase synchrony are due to experts’ enhanced fronto-parietal connectivity related to either solely on the activation of the multiple demand system (Duncan 2010), but more likely the interaction of the multiple-demand and domain-specific visuospatial processes (Amalric and Dehaene 2016) when mentally processing long advanced mathematics.

In addition to slow wave sleep (e.g. Hofle et al. 1997; Fultz et al. 2019), recently delta oscillation has been shown to increase during concentrated meditative states (Yordanova et al. 2020; Volodina et al. 2021). Delta oscillations are implicated in the inhibition of neural processing, with increased delta synchrony associated with increased inhibition in sensory cortices (Harmony 2013 for a review). However, such inhibition is proposed to have two different functions. According to Vogel et al. (1968) and further reflected by Harmony (2013), “Class I inhibition” refers to a gross inactivation of an entire excitatory process resulting in a less active state, as in sleep. “Class II inhibition” selectively suppresses inappropriate or non-relevant neural activity during the performance of a mental task, including disturbing sensory stimuli and task-irrelevant thoughts. Our results may present enhanced Class II inhibition in experts in comparison to novices, which is in line with enhanced engagement during math demonstrations that experts reported in our study. Further, Class II inhibition may facilitate the functional fronto-parietal connectivity in experts and optimize the interaction of the multiple-demand and domain-specific processes during long advanced mathematics. However, it will be crucial to study this interpretation further with fMRI due to its precise spatial resolution, and to investigate the possibility that the activation of the multiple-demand system would solely explain the group differences in our study.

Our results showing a more wide-spread enhancement in delta phase synchrony in experts than novices after two seconds from the stimulus onset suggest that long and complex math tasks require a close collaboration between frontal and parietal regions. Over the first two seconds, which is a similar duration as the tasks in conventional math studies (e.g. De Smedt et al. 2009; Grabner and De Smedt 2012; Kulasingham et al. 2021), the differences between experts and novices on delta phase synchrony exist to lesser extent than from two seconds onwards from the stimulus onset (Fig. 5b). For novices, it might be harder to enter in such a deep internal concentration due to math demonstrations which they reported not to understand so well, not being as engaged to and not having enough of time to solve them in comparison to the self-evaluation reports of experts. On the other hand, deeper engagement and concentration of experts may facilitate the interactions of the large networks, including multiple-demand and visuospatial networks, during the cognitively demanding task of advanced mathematics, leading to faster processing and better understanding of the demonstrations.

### Body posture and cognition

We also found that experts had a stronger delta phase synchrony over bilateral fronto-parietal electrodes than novices when standing (Fig. 6a and 6b). Previous studies with simple cognitive tasks have shown that body posture modifies cognitive functions (Awad et al. 2021), and therefore, we wanted to explore the influence of the body posture also to advanced mathematical processing. For example, standing is shown to enhance visuospatial working memory in comparison to sitting (Dodwell et al. 2019), and since visuospatial network is crucial in mathematical processing (Amalric and Dehaene 2016; Wang et al. 2022), its functions may be modified differently in math experts and novices when sitting or standing.

Elevated arousal state when standing in comparison to sitting may influence differently in the function of the extended multiple-demand system in math experts and novices during advanced mathematical processing (Camilleri et al. 2018). Another aspect, which we did not consider in our explorative hypothesis on body posture, is the dual-task attentional demands the standing body posture combined with mathematical problem solving may evoke. Standing seems to compromise performance on problems requiring a burst of insight in comparison to sitting (Lipnicki and Byrne 2005). In addition, sustained upright posture requires constant attentional engagement (Woollacott and Shumway-Cook 2002). In our study, potential dual-task attentional demands between body balance and math processing may challenge more math novices than experts, who are shown to have automatic mathematical processing possibly requiring less attentional efforts (Jeon et al. 2019). Thus, there may be a trade-off with the potentially enhanced functioning of the extended multiple domain system in standing body posture and the increased attentional demand such body posture may cause, especially when engaging with cognitively challenging tasks with low prior knowledge, like when math novices process bachelor-level mathematics.

### Limitations

There are a few limitations to discuss. First, common issue with the phase synchrony method is volume conduction which means that signal from two neighboring EEG electrodes is influenced by the same brain functions instead of separate brain functions in synchrony. Therefore, it is likely that delta phase synchrony reported in near EEG electrode pairs, like Pz—P2 and P2—PPO1, are contaminated by volume conduction. However, we also found delta phase synchrony between distant electrode pairs including frontal, central and parietal locations, like F3—P1, F3—Pz and F3—PPO1, suggesting expertise-related differences in functional connectivity between distant brain regions. Volume conduction is a severe issue over near but not over distant electrodes. Second, our results could be explained by fundamentally different brain functions between experts and novices assuming that experts followed the math demonstrations, but novices were just thinking other task-irrelevant thoughts. Indeed, novices reported being less engaged and having a poorer understanding of the demonstrations than experts. However, we can assume that also novices did their best to understand the demonstrations because all the participants knew they would be asked to explain the demonstrations later to the experimenter. Third, despite we aimed for age-matched groups, due to technical problems and rejection of several participants, the age of the expert and novice groups differed statistically. The mean age of math experts was 21.0 years of novices 23.8 years. There are no comparative studies on the neural correlates of mathematical cognition in participants in early twenties and we assume that the age difference, despite statistically significant, does not explain the group differences in theta phase synchrony. Generally, differences in brain functions related to cognition with an age gap of 3–4 years can be found in children and adolescents (e.g. Arain et al. 2013), and on the other hand with larger age gaps, between young adults, middle-aged people and elderly (e.g. Cansino et al. 2015). Fourth, our study did not include other cognitively challenging task than mathematics, unlike previous studies on expertise and advanced mathematics (Amalric and Dehaene 2016, 2019; Wang et al. 2022). Thus, we cannot know whether the cortical connectivity results of our study are explained solely by the activation of the multiple-demand system (Duncan 2010), extended multiple demand-system (Camilleri et al. 2018), or the interaction of the multiple-demand and domain-specific visuospatial processes. In the light of previous expertise studies (Amalric and Dehaene 2016, 2019), we suggest that the fronto-parietal cortical synchrony is explained by the latter interaction. However, a future fMRI-EEG study with long advanced mathematics and other cognitively challenging task, like history, which are matched with their difficulty level is required. Ideally, in such study the participant groups would include experts in mathematics, experts in the other chosen discipline, like history, and people who are novices in both disciplines. Only then, we could pinpoint the brain networks underlying the fronto-parietal delta synchrony observed in our study. Lastly, it is important to note that, unlike in the conventional math studies conducted with short stimuli, the duration of the stimuli (math demonstrations) in our study varied between the demonstrations. However, we considered this as a reasonable trade-off when being able to study the brain with longer and more complex stimuli than the ones conventionally used in neuroscientific math studies.

## Conclusion

In summary, we have demonstrated that math experts have enhanced delta phase synchrony over fronto-parietal electrodes during long advanced mathematics in comparison to novices. These results suggest enhanced functional connectivity between multiple-demand and domain-specific visuospatial functions in experts. Our study has a novelty value for neurosciences of mathematical cognition in three aspects, since we 1) expanded the frequencies studied to lower delta band and inter-regional synchronies, 2) used long advanced mathematics as stimuli, and 3) studied mathematical processing in different body postures. We aimed to maintain some of the controllability similar to the traditional reductionist study settings by conducting the study in an EEG laboratory and utilizing time stamping of the presentation of the math tasks and participants’ self-evaluations on engagement and understanding after each math task. However, we simultaneously aimed to use a naturalistic, real-world like, aspect in our study design by showing participants long and complex math demonstrations, with a difficulty level comparable to early university studies which take up to one minute to solve, and by exploring the mathematical cognition when sitting and standing.

Future empirical and theoretical work should be directed to understand more in detail the mechanisms by which interaction between multiple-demand and domain-specific processes occur, to which extend expertise and internal concentration facilitate such interaction, and in which way the networks are recruited during different timepoints of complex mathematical tasks. Source localization would be an adequate complementing analysis for the signal-level analysis to locate the origins of the dynamical cortical oscillations related to math expertise and processing of long and complex math demonstrations. Our results encourage to use longer and more complex math tasks and different body postures in the future studies to reach towards understanding the brain functions related to mathematics solved in the real life. By investigating the brain functions in naturalistic settings, with engagement, and expertise, we can also understand better the cerebral processes underlying the gradual transformation from novice to expert.

## Data Availability

The data will be openly published in autumn 2024, and until then, the data can be received by requesting them from the corresponding author.
